# The dual HCK/BTK inhibitor KIN-8194 impairs growth and integrin-mediated adhesion of BTKi-resistant mantle cell lymphoma

**DOI:** 10.1038/s41375-024-02207-9

**Published:** 2024-03-07

**Authors:** Hildo C. Lantermans, Fangxue Ma, Annemieke Kuil, Sanne van Kesteren, Sevtap Yasinoglu, Guang Yang, Sara J. Buhrlage, Jinhua Wang, Nathanael S. Gray, Marie José Kersten, Steven P. Treon, Steven T. Pals, Marcel Spaargaren

**Affiliations:** 1 Department of Pathology, Amsterdam UMC location University of Amsterdam, Meibergdreef 9, Amsterdam, The Netherlands; 2 Lymphoma and Myeloma Center Amsterdam – LYMMCARE, Amsterdam, The Netherlands; 3 Cancer Center Amsterdam (CCA), Cancer Biology and Immunology - Target & Therapy Discovery, Amsterdam, The Netherlands; 4 Bing Center for Waldenström Macroglobulinemia, Dana-Farber Cancer Institute, Boston, MA USA; 5 Department of Medical Oncology, Dana-Farber Cancer Institute and Harvard Medical School, Boston, MA USA; 6 Department of Cancer Biology, Dana-Farber Cancer Institute, and Department of Biological Chemistry and Molecular Pharmacology, Harvard Medical School, Boston, MA USA; 7 Department of Chemical and Systems Biology, ChEM-H, Stanford Cancer Institute, School of Medicine, Stanford University, Stanford, CA USA; 8 Department of Hematology, Amsterdam UMC location University of Amsterdam, Amsterdam, The Netherlands; 9 Present Address: Blueprint Medicines, Cambridge, MA USA

**Keywords:** B-cell lymphoma, Cancer microenvironment, Cancer therapeutic resistance

## Abstract

Although Bruton’s tyrosine kinase (BTK) inhibitors (BTKi) have significantly improved patient prognosis, mantle cell lymphoma (MCL) is still considered incurable due to primary and acquired resistance. We have recently shown that aberrant expression of the Src-family tyrosine kinase hematopoietic cell kinase (HCK) in MCL correlates with poor prognosis, and that genetic HCK perturbation impairs growth and integrin-mediated adhesion of MCL cells. Here, we show that KIN-8194, a dual inhibitor of BTK and HCK with in vivo activity against Myd88-L265P-driven diffuse large B-cell lymphoma and Waldenström Macroglobulinemia, has a potent growth inhibitory effect in MCL cell lines and primary MCL cells, irrespective of their sensitivity to BTKi (ibrutinib and acalabrutinib). In BTKi-resistant cells this is mediated by inhibition of HCK, which results in repression of AKT-S6 signaling. In addition, KIN-8194 inhibits integrin-mediated adhesion of BTKi-sensitive and insensitive MCL cells to fibronectin and stromal cells in an HCK-dependent manner. Finally, we show that MCL cells with acquired BTKi resistance retain their sensitivity to KIN-8194. Taken together, our data demonstrate that KIN-8194 inhibits growth and integrin-mediated adhesion of BTKi-sensitive MCL cells, as well as MCL cells with primary or acquired BTKi resistance. This renders KIN-8194 a promising novel treatment for MCL patients.

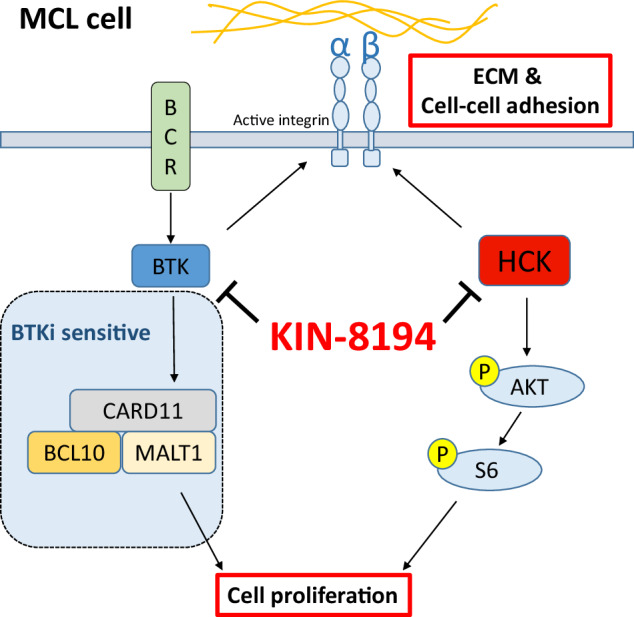

## Introduction

Mantle cell lymphoma (MCL) is an aggressive subtype of non-Hodgkin lymphoma with a poor clinical outcome, arising from B-cells in the mantle zone surrounding germinal centers. MCL is characterized by the t(11;14)(q13;q32) translocation, resulting in overexpression of the cell-cycle regulator cyclin D1. Although advances in MCL therapy have improved the prognosis of patients with MCL, treatment typically is not curative [1].

Among the novel therapeutic options are BTK inhibitors (BTKi), such as ibrutinib, acalabrutinib, and zanubrutinib which act on the BCR signaling pathway. We have previously shown that BTKi have a unique molecular mechanism besides directly reducing MCL growth: by inhibiting the integrin-mediated adhesion of MCL cells to the lymphoid microenvironment the malignant cells are mobilized into the peripheral blood, which deprives the MCL cells from critical growth and survival factors provided in the lymphoid organs and bone marrow [2–5]. Although the use of BTKi has improved MCL patient outcome, one-third of patients show primary resistance and the emergence of secondary resistance eventually seems to be inevitable [6–8]. Various resistance mechanisms to BTKi have been described, including upregulation of the PI3K-AKT pathway, activation of non-canonical NF-κB signaling, and mutations in BTK and CARD11 [9–16].

The SRC-family tyrosine kinase (SFK) hematopoietic cell kinase (HCK), which is predominantly expressed in the hematopoietic cell lineage, has been implicated in various cellular processes including inflammation, proliferation and actin polymerization [17–20]. We have recently shown that HCK is aberrantly upregulated in MCL patients and that high HCK expression correlates with poor clinical outcome [21]. In addition, patients with high HCK expression show strong enrichment of gene-sets involved in TLR signaling, and HCK expression in MCL is driven by TLR-MYD88 signaling [21]. Furthermore, genetic silencing of HCK in MCL cell lines resulted in inhibition of growth and integrin-mediated adhesion to fibronectin or stromal cells [21].

Recently, KIN-8194, a dual inhibitor of BTK (IC50 = 0.915 nM) and HCK (IC50 < 0.495 nM), was shown to potently affect the survival of ABC-type DLBCL and Waldenström Macroglobulinemia with a pathogenic Myd88-L265P mutation, while minimally affecting healthy B-cells [22]. Importantly, KIN-8194 showed excellent pharmacokinetic and pharmacodynamics properties in vivo and was well tolerated in mice [22]. Furthermore, as compared to treatment with ibrutinib, KIN-8194 treatment resulted in superior survival of mice xenografted with the ABC-DLBCL cell line TMD8 [22]. Lastly, the effects of KIN-8194 were not affected by the BTK^C481S^ mutation, which has been frequently detected in patients with acquired BTKi resistance [22].

Our previous characterization of both BTK and HCK as suitable targets in MCL led us to investigate the therapeutic potential of KIN-8194 in MCL. We show that KIN-8194 potently inhibits the growth of MCL cells, irrespective of their sensitivity to BTKi, which is mainly mediated by HCK inhibition. Furthermore, KIN-8194 inhibits the adhesion of all MCL cells to fibronectin or stromal cells whereas ibrutinib exclusively affects the BTKi-sensitive cells. Lastly, we show that KIN-8194 treatment potently reduces the growth of MCL cells with acquired resistance to BTKi. Taken together, our data demonstrate that KIN-8194 has added value over BTKi in MCL preclinical models.

## Materials and methods

### Cell culture

MCL cell lines JeKo-1, Mino, Rec-1, Granta-519, Maver-1, and Z-138 (all from DSMZ) were cultured in IMDM/10% FCS/2mM L-glutamine/100 units/ml penicillin/100 µg/ml streptomycin and stromal cell lines HS-5 and HS-27a (both from ATCC) in DMEM/10% FCS/2 mM L-glutamine/100 units/ml penicillin/100 µg/ml streptomycin. JeKo-R cells were generated by prolonged (~6 months) culture of JeKo-1 cells with 100 nM Ibrutinib. Cell lines were routinely authenticated by STR-profiling (Promega) and verified for mycoplasma contaminations.

Peripheral blood-derived MCL cells were obtained after routine diagnostics or follow-up procedures at our Department of Hematology (Amsterdam UMC, location UvA) and purified using Ficoll and B-cell isolation kit (Miltenyi Biotec). MCL cells with less than 90% CD5^+^/CD19^+^ were sorted on a BD-FACS-AriaIIu. Primary MCL cells were cultured on irradiated 3T3-CD40L cells in RPMI/20% FCS/2 mM L-glutamine/100 units/ml penicillin/100 µg/ml streptomycin. This study was approved by the AUMC Medical Committee on Human Experimentation and conducted in accordance with the revised Declaration of Helsinki 2008.

### Cloning and transduction

LZRS-HCK^T333M^-IRES-GFP was constructed by cloning HCK cDNA into LZRS-IRES-GFP (provided by Dr. H. Spits), and threonine-333 was mutated to methionine with the QuickchangeIIXL site-directed mutagenesis kit (Agilent Technologies). The mutation was confirmed by Sanger sequencing. LZRS-BTK^C481S^-IRES-GFP was constructed by subcloning from pLenti-IRES-BTK^C481S^-GFP. pMSCV-CARD11^D357E^-puro was constructed by mutating pMSCV-CARD11-puro (provided by Dr. G. Lenz) with the QuickchangeIIXL site-directed mutagenesis kit. The mutation was confirmed by Sanger sequencing. Retroviral particles were generated by transfecting constructs into Phoenix-Galv cells with Genius DNA transfection reagent (Westburg). Harvested retroviral particles were bound to retronectin-coated plates by centrifugation for 2 h at 3250 × *g*. MCL cells were added and three days after transduction GFP-sorted on a SH800 Cell Sorter (Sony). Mino-CARD11^D357E^ cells were selected for 4 days with 4 µg/ml puromycin followed by functional selection with 100 nM ibrutinib for 7 days. Mino-Cas9^CARD11-Δ350-355^ cells were generated by transfecting Mino-Cas9 cells with pLenti-guide-puro containing a guide targeting CARD11 (CAAGTGCTCGACCCTGGGAA) and a donor template (TACCTGGAGGAGAAGGAGGACCTGGAGCTCAAGTGCTCGACCCTGGGAAAAGAATGTGAAATGTACAAGCACCGCATGAACACGGTCATGCTGCAGCTGGAGGAGGT) with the Neon transfection system (1700 V/20 ms/1×) followed by selection with 2 µg/ml puromycin for 2 days and subsequent functional selection with 100 nM ibrutinib for 14 days. In the surviving cells we identified the deletion in CARD11 by Sanger sequencing.

### Growth experiments

MCL cells were seeded at 3000 cells per well with the indicated amount of inhibitor in flat-bottom 96-well plates in IMDM/10% and cultured for 7 days at 37 °C, 5% CO_2_. For experiments involving Mino-Cas9^CARD11-Δ350-355^, Mino^CARD11-D357E^, or the appropriate controls 1500 cells were seeded. After 7 days 100 ng/ml of the viability dye 7-AAD (Invitrogen) was added to each well and incubated for 30 min at 37 °C, 5% CO_2_ after which the number and percentage of viable cells was determined by flow cytometry. The number of viable cells were normalized to the relevant DMSO control, which was set to 100%.

For experiments with primary MCL first 75,000 lethally irradiated 3T3 cells expressing CD40L (3T3-CD40L) were seeded in flat-bottom 96-well plates and a total of 50,000 primary MCL cells were added, followed by the indicated amount of inhibitor. Primary cells were incubated for 5 days at 37 °C, 5% CO_2_ after which ATP-activity was determined with Celltiter-Glo (Promega) according to the manufacturer’s instructions. In parallel, primary MCL cells were stained with 100 nM of the viability dye To-Pro-3 (Invitrogen) and the percentage of viable primary MCL cells was determined by flow cytometry. The ATP-activity and percentage of viable primary MCL cells were normalized to the relevant DMSO control, which was set to 100%.

### BrdU cell-cycle analysis

For cell-cycle analysis, cells were incubated for 1 h with 20 µM BrdU (Sigma-Aldrich), washed once with PBS/0,1% BSA, and subsequently fixed in ice-cold 75% ethanol/PBS. After washing, the cells were incubated with 0,4 mg/ml pepsin in 0.1 mM HCl for 30 min at room temperature and subsequently with 2 M HCl for 25 min at 37 °C. Cells were washed once with PBT (PBS/0,05% Tween-20) and once with PBTB (PBT/2% BSA) and stained for 30 min with anti-BrdU-FITC (clone B44, BD Biosciences) in PBTB. After washing with PBT and PBTB, cells were treated with 500 µg/ml RNAse A (Qiagen) and stained with 100 nM To-Pro-3 (Invitrogen) in PBS/1% BSA for 15 min at 37 °C and analyzed by flow cytometry.

### Immunoblotting

For immunoprecipitation 10 × 10^6^ cells were treated with the indicated concentration of inhibitor for 30 min after which cells were lysed in 500 µl RIPA buffer. After the removal of cellular debris by centrifugation (30 min, 14,000 × *g*, 4 °C) the lysates were precleared while rotating for 60 min at 4 °C with 20% protein A sepharose beads to exclude proteins that bind to protein A sepharose beads. After centrifugation (30 s, 11,000 × *g*, 4 °C) the cleared supernatants were incubated overnight, while rotating, with an antibody against HCK (1:200, CST, #14643) followed by one hour with the addition of 20% protein A sepharose beads. Beads were collected by centrifugation (30 s, 11,000 × *g*, 4 °C) and washed 4 times with lysisbuffer, followed by elution with sample buffer and immunoblotting. Controls either lack cells (not shown) or anti-HCK, and were analyzed on the same blot or a parallel blot with equal exposure time. To prevent obstruction of the HCK signal by the Ig heavychain (~50 kDa), the PVDF membrane was carefully trimmed until the Ig heavychain was no longer detected. All protein samples were separated on Bolt™ 4–12% Bis-Tris Plus gels (Invitrogen) and subsequently blotted to a PVDF membrane. Antibodies: anti-p-BTK(Y223) (CST, #5082), anti-BTK (BD, #611116), anti-phospho-tyrosine (Sigma, 4G10), anti-p-AKT(S473) (CST, #4060), anti-pan-AKT (CST, #4691), anti-p-S6(S325/S326) (CST,#4858), anti-S6 (CST, #2217), anti-HCK (CST, #14643), anti-CYLD (total and 70 kDa, E-10 Santa Cruz), anti-CYLD (40 kDa, #8462 CST), anti-CARD11 (CST, #4435) and anti-β-actin (Sigma-Aldrich, A1978). Primary antibodies were detected with anti-mouse-HRP or anti-rabbit-HRP (both DAKO).

### Adhesion experiments

Adhesion assays were performed as previously described [3, 23]. Assays on fibronectin were performed on 96-well Microlon® high binding plates (Greiner), coated overnight with PBS containing 10 µg/ml (JeKo-1, Maver-1) or 0,67 µg/ml (Granta-519) fibronectin (Sigma-Aldrich) at 4 °C. On the day of the experiment plates were blocked for 1 h at 37 °C with IMDM/4% BSA. For adhesion to stromal cells, 15,000 HS-27a-GFP or 50,000 HS-5-GFP cells were seeded in a tissue culture-treated 96-well plate one day prior to the experiment. MCL cells were pretreated for 30 min with inhibitors at 37 °C while rotating and 150,000 cells were seeded per well, with or without PMA (50 ng/ml) or αIgM (0,5 µg/ml), and incubated at 37 °C, 5% CO_2_ for 30 min. For primary MCL, 500,000 cells were seeded per well. For adhesion to fibronectin, after washing with IMDM/0,5% BSA to remove non-adherent cells, the cells were fixed for 10 min with 10% glutaraldehyde/PBS (Millipore) and stained with 0,4% crystalviolet/20% ethanol. After washing with ddH_2_O, the dye was eluted with ethanol and quantified by measuring absorbance at 570 nm (Clariostar, BMG-Labtech). Nonspecific adhesion to wells coated with 4% BSA was subtracted. For adhesion to stromal cells, after washing with IMDM/0,5% BSA, cells were trypsinized and quantified by flow cytometry. To account for the loss of stromal cells the ratio between MCL cells and stromal cells was determined.

### Flow cytometric analysis

Following incubation for 30 min with the indicated concentration of inhibitor, 10^5^ cells were stained with anti-α4-integrin (HP2/1, Millipore), anti-β1-integrin (4B4, Coulter Immunology), anti-β2-integrin (IB4, Santa Cruz) or the respective control, followed by staining with PE-conjugated anti-mouse IgG1 or PE-conjugated anti-mouse IgG2. Cell surface staining was analyzed by flow cytometry.

## Results

### KIN-8194 inhibits the growth of MCL cell lines and primary cells

**Fig. 1:**
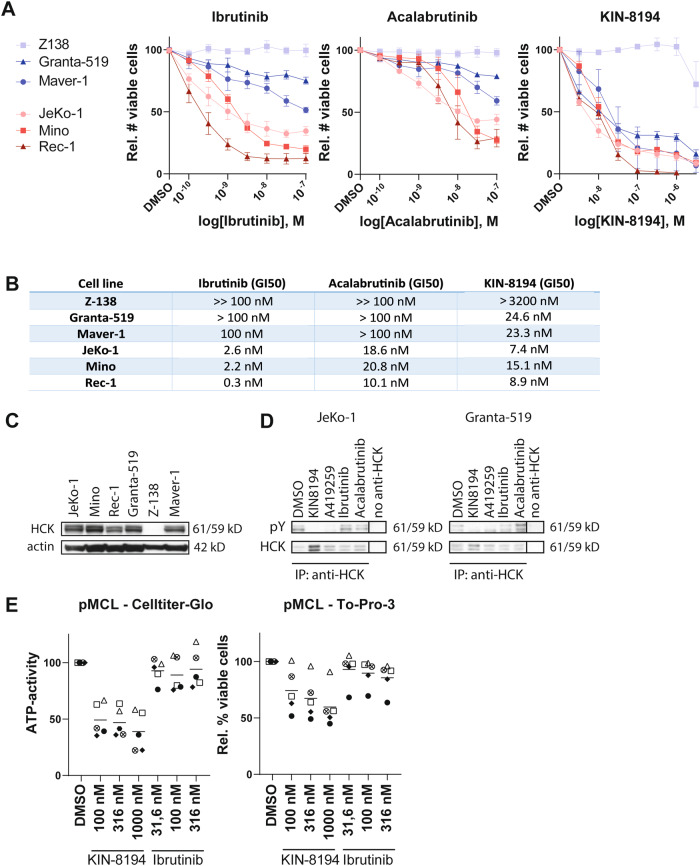
KIN-8194 inhibits the growth of BTKi-sensitive and insensitive MCL cells. **A** Number of viable MCL cells, determined by flow cytometric analysis and 7-AAD staining after 7 days of treatment with various concentrations of ibrutinib, acalabrutinib, or KIN-8194. The number of viable cells was normalized to the DMSO-treated condition. Data are presented as mean ± S.E.M. of at least three independent experiments performed in triplicate. **B** GI50 of KIN-8194, ibrutinib, and acalabrutinib as determined by interpolating the concentration required to achieve 50% of the DMSO-treated cells from a non-linear regression curve. **C** Western blot analysis of HCK expression in MCL cell lines. Actin serves as a loading control. **D** Western blot analysis of HCK phosphorylation. JeKo-1 and Granta-519 cells were treated for 30 min with 100 nM KIN-8194, A-419259, ibrutinib, or acalabrutinib at 37 °C. HCK was immunoprecipitated from cell lysates and immunoblotted with an anti-phosphotyrosine (pY) antibody, followed by an anti-HCK antibody to assess immunoprecipitation efficacy. The pY bands are at the same molecular weight as the observed HCK bands and are absent in the control immunoprecipitations without anti-HCK. **E** ATP-activity of 5 primary MCL samples as determined by CellTiter-Glo assay, and the percentage of viable primary MCL cells as determined by To-Pro-3 staining, after 5 days of treatment with various concentrations of KIN-8194 or ibrutinib. ATP-activity and cell viability were normalized to the DMSO-treated condition.

To study the therapeutic potential of KIN-8194 for MCL treatment, we assessed its effect on cell growth, in comparison with clinically applied BTK inhibitors (BTKi), in a panel of MCL cell lines. It has previously been shown that some MCL cell lines are sensitive to BTKi, whereas others are insensitive [13, 24]. In line with these findings the BTK inhibitors ibrutinib and acalabrutinib reduced the growth of MCL cell lines JeKo-1, Mino-1, and Rec-1, but hardly affected the growth of Z-138, Maver-1, and Granta-519 even though BTK is expressed and active in these cell lines (Fig. 1A, Supplementary Fig. 1A). In contrast, KIN-8194 potently reduced the growth of all studied MCL cell lines (GI_50_: 7–25 nM), with the exception of Z-138 (Fig. 1A, B), an atypical MCL cell line with blastoid transformation and the only tested MCL cell line that lacks HCK expression (Fig. 1C) [21, 25]. The viability of the MCL cell lines, with the exception of Rec-1, was not affected by BTKi or KIN-8194, indicating that the observed effects are the result of inhibition of cell proliferation (Supplementary Fig. 1B). Indeed, cell-cycle analysis showed that treatment of MCL cell lines with KIN-8194 resulted in an increase of cells in G1 and a decrease of cells in S-phase (Supplementary Fig. 1C). In accordance, we have previously demonstrated that, except for Z-138, HCK is aberrantly expressed in all analyzed MCL cell lines as well as 6 out of 7 tested primary MCL patient samples, and that genetic silencing of HCK in JeKo-1 and Granta-519 inhibits their proliferation by G1 arrest [21]. Furthermore, treatment of JeKo-1 and Granta-519 with 100 nM KIN-8194 or the SFK inhibitor A-419259, but not with BTKi, reduced the phosphorylation of HCK (Fig. 1D).

In primary MCL cells, co-cultured with CD40L-expressing fibroblasts, KIN-8194 reduced the ATP-activity in all 5 tested patient samples, with a mean inhibition of 51% after treatment with 100 nM KIN-8194, whereas ibrutinib treatment only marginally affected 3 out of 5 samples, even at a relatively high concentration of 316 nM (Fig. 1E). Furthermore, the percentage of viable cells was reduced in 4 out of 5 tested primary MCL patient samples by KIN-8194 treatment, whereas ibrutinib only affected 1 out of 5 samples. Since the primary MCL cells did not proliferate uniformly, it was not possible to assess the growth inhibitory effect of KIN-8194. Please note that these tested MCL samples all express HCK [21]. Taken together, these results demonstrate that KIN-8194 inhibits the proliferation of BTKi-sensitive and -insensitive MCL cell lines, with the exception of the HCK-negative atypical MCL cell line Z-138, and also impedes the ATP-activity and viability of primary MCL samples, whereas ibrutinib only had modest effects.

### Inhibition of HCK by KIN-8194 reduces MCL proliferation

In order to assess the role of HCK in the effects of KIN-8194, we overexpressed the HCK^T333M^ gatekeeper mutant, which renders the ATP binding site inaccessible to inhibitors, in the KIN-8194 sensitive MCL cell lines Granta-519, Mino, JeKo-1 and Maver-1 (Supplementary Fig. 2A). Overexpression of HCK^T333M^ did not affect the growth of the tested MCL cell lines (Supplementary Fig. 2B). Please note that, in contrast to ectopic overexpression of wildtype HCK, HCK^T333M^ overexpression does not result in sequestering of inhibitors, which would compromise their effect on other kinases.

**Fig. 2:**
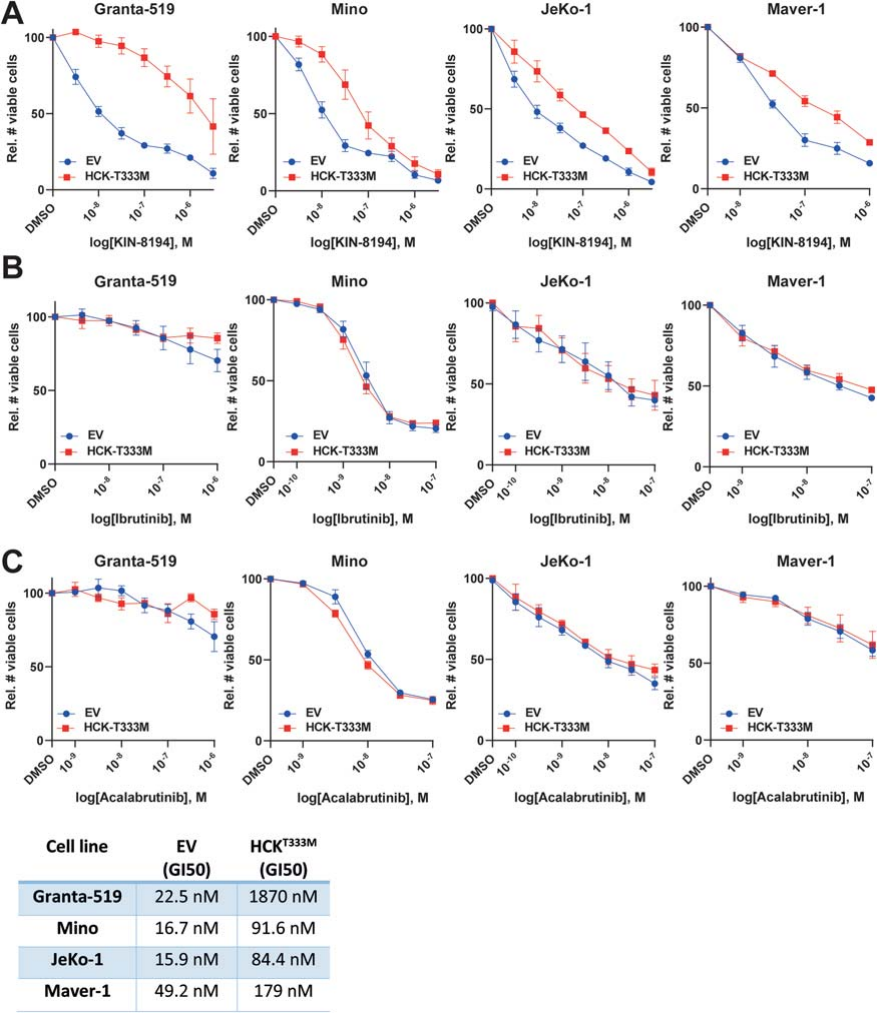
HCK-mediated growth inhibition by KIN-8194 and ibrutinib. **A**–**C** The number of viable Granta-519, Mino, JeKo-1, and Maver-1 cells transduced with LZRS-GFP empty vector (EV) control or LZRS-HCK^T333M^-GFP after 7 days of treatment with various concentrations of KIN-8194 (**A**), ibrutinib (**B**), or acalabrutinib (**C**), as determined by flow cytometric analysis and 7-AAD staining. The number of viable cells was normalized to the DMSO-treated condition. Data are presented as mean ± S.E.M. of at least three independent experiments performed in triplicate.

In the BTKi-insensitive cell line Granta-519 overexpression of HCK^T333M^ drastically reduced the growth inhibitory potency of KIN-8194, lowering the GI50 more than 75-fold (Fig. 2A). In Mino, JeKo-1 and Maver-1, HCK^T333M^ overexpression also reduced the growth inhibitory effect of KIN-8194, but to a lesser extent (3.5–5 fold) (Fig. 2A). Thus, the effects of KIN-8194 on the growth of Granta-519 is largely due to HCK inhibition whereas in JeKo-1, Mino, and Maver-1 KIN-8194 acts via inhibition of multiple kinases including HCK. Since ibrutinib, but not acalabrutinib, has been shown to also inhibit HCK [26–28], we assessed the effect of HCK^T333M^ overexpression on growth inhibition by both BTKi. HCK^T333M^ overexpression did not affect the potency of either ibrutinib or acalabrutinib in the tested MCL cell lines (Fig. 2B, C), indicating that the growth effects of neither BTKi is mediated via HCK inhibition.

### KIN-8194 abrogates AKT/S6 signaling in BTKi-insensitive MCL via HCK

**Fig. 3:**
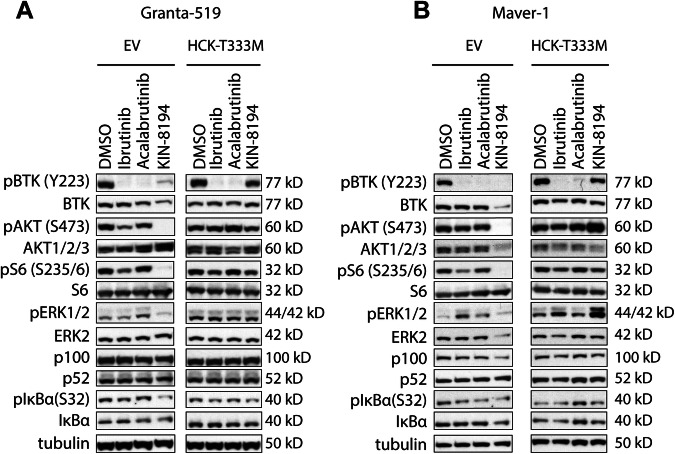
KIN-8194 inhibits the AKT-S6 signaling pathway in an HCK-dependent manner. Western blot analysis of Granta-519 (**A**) and Maver-1 (**B**) cells transduced with LZRS-GFP empty vector (EV) control or LZRS-HCK^T333M^-GFP, treated with 100 nM of KIN-8194, ibrutinib or acalabrutinib for 6 h. Tubulin serves as a loading control.

To explore the signaling pathways inhibited by KIN-8194 in BTKi-insensitive cells and the contribution of HCK inhibition therein, we analyzed the phosphorylation of various signaling molecules in Granta-519 and Maver-1 cells transduced with HCK^T333M^ upon treatment with KIN-8194 or BTKi. In Granta-519 and Maver-1 nanomolar concentrations of KIN-8194 inhibited phosphorylation of AKT and S6, a downstream mediator of AKT-mTOR signaling (Fig. 3 and Supplementary Fig. 3). HCK^T333M^ overexpression In Maver-1 completely rescued the effects of KIN-8194 on AKT and S6 phosphorylation, and in Granta-519 an almost complete rescue was observed, even at the highest concentrations tested (Fig. 3 and Supplementary Fig. 3). In contrast, in both Maver-1 and Granta-519 ibrutinib treatment only partially reduced phosphorylation of AKT and S6 (Fig. 3 and Supplementary Fig. 3). Since these effects were overcome by HCK^T333M^ overexpression, this partial reduction in AKT and S6 phosphorylation is due to HCK inhibition by ibrutinib (Fig. 3 and Supplementary Fig. 3). In line with this, acalabrutinib treatment, which does not target HCK, did not result in reduced AKT or S6 phosphorylation (Fig. 3). Notably, HCK^T333M^ overexpression did not rescue the growth inhibitory effect of ibrutinib in Granta-519 and Maver-1 (Fig. 2), indicating that the partial repression of AKT/S6 signaling upon HCK inhibition by ibrutinib is not sufficient to reduce proliferation in these cells. KIN-8194 treatment only weakly affected other established mediators of cell proliferation, such as the MAPK (pERK1/2), and canonical (pIκBα and IκBα) or non-canonical Nf-κB (p100/p52) signaling pathways (Fig. 3 and Supplementary Fig. 3). Please note that the reduction in BTK levels upon KIN-8194 treatment in Fig. 3 was not consistent across experiments (see also Supplementary Fig. 3 and, for Mino, Fig. 5D, F), and is therefore considered not representative. Together, these findings suggest that KIN-8194 inhibits the growth of BTKi-insensitive MCL cell lines by completely abrogating the AKT-S6 signaling pathway in an HCK-dependent manner. In support of this, treatment of all MCL cell lines with the AKT inhibitor MK-2206 or the MTOR inhibitor rapamycin resulted in growth inhibition without affecting the cell viability, similar to KIN-8194 treatment (Supplementary Fig. 4).

### KIN-8194 inhibits integrin-mediated adhesion of MCL cells irrespective of sensitivity to BTKi

**Fig. 4:**
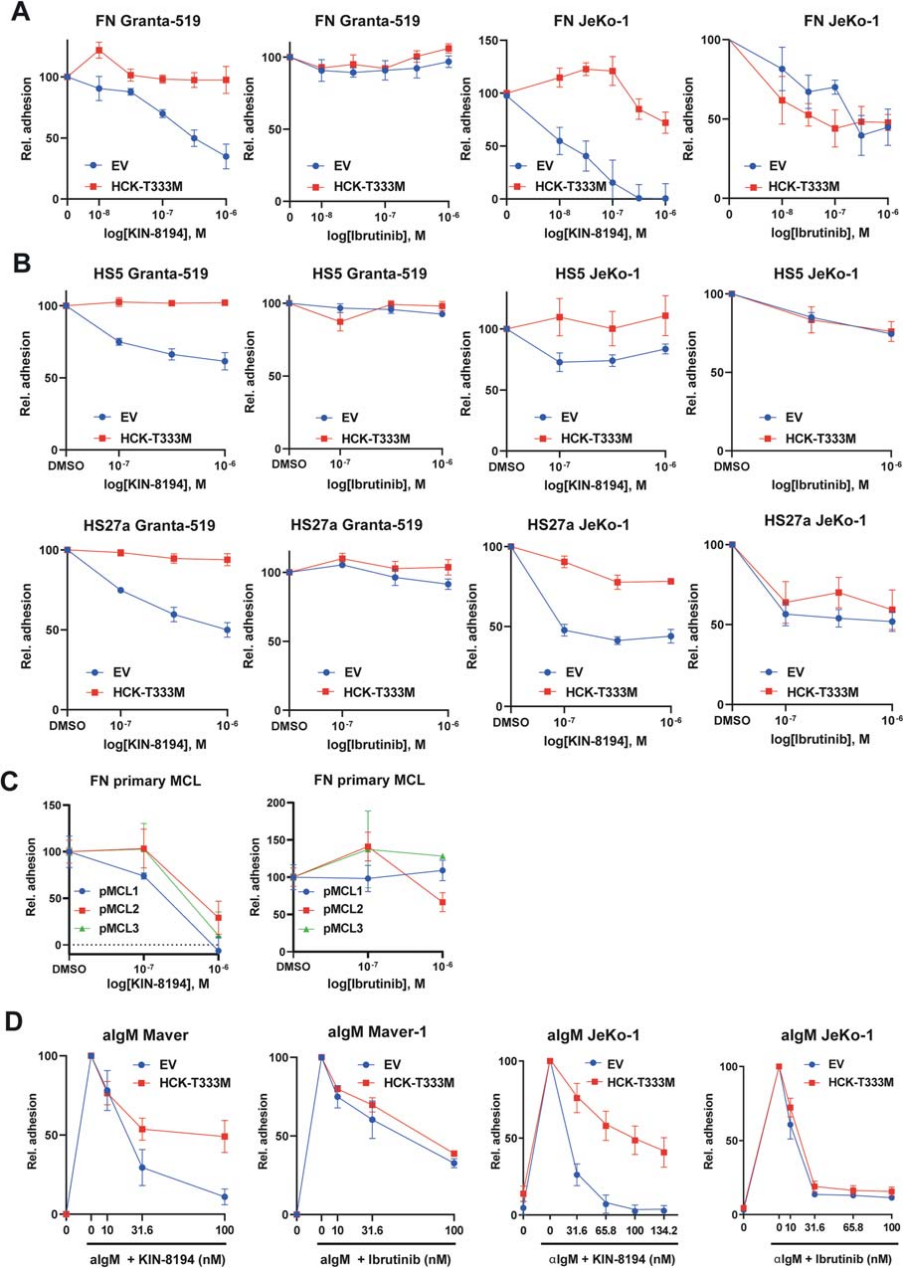
KIN-8194 inhibits adhesion of MCL cells to fibronectin or stromal cells in an HCK-dependent manner. Adhesion of Granta-519 and JeKo-1 transduced with LZRS-GFP empty vector (EV) control or LZRS-HCK^T333M^-GFP, treated for 30 min with various concentrations of KIN-8194 or ibrutinib to fibronectin (**A**) or stromal cells HS-5 and HS-27a (**B**). Adhesion to fibronectin of primary MCL cells treated for 30 min with various concentrations of KIN-8194 or ibrutinib (**C**). Adhesion to fibronectin of Maver-1 and JeKo-1 cells transduced with LZRS-GFP empty vector (EV) control or LZRS-HCK^T333M^-GFP, treated for 30 min with various concentrations of KIN-8194 or ibrutinib and stimulated with aIgM (**D**). Cells were allowed to adhere for 30 min. Non-adherent cells were removed by washing. For adhesion to stromal cells the ratio between MCL cells and stromal cells was determined. The percentage of adhesion was normalized to the untreated control. Data are presented as mean ± S.E.M. of at least three independent experiments performed in triplicate.

Given the importance of targeting integrin-mediated retention of MCL cells in the lymphoid organ microenvironment for the clinical efficacy of ibrutinib [2–4], we next investigated whether KIN-8194 inhibits the integrin-mediated adhesion of MCL to fibronectin (FN) and the stromal cell lines HS-5 and HS-27a. KIN-8194 pretreatment reduced the adhesion of Granta-519 to FN, HS-5, and HS-27a in a dose-dependent manner (Fig. 4A, B). KIN-8194 treatment also inhibited the adhesion of Maver-1 to HS-27a but not to HS-5 (Supplementary Fig. 5). Since the adhesion of Maver-1 to FN was very weak the effect of KIN-8194 on adhesion of Maver-1 to FN could not be assessed. Overexpression of HCK^T333M^ completely overcame the KIN-8194-induced loss-of-adhesion of Granta-519 and Maver-1, indicating that these effects are HCK-mediated (Fig. 4A, B and Supplementary Fig. 5). Overexpression of HCK^T333M^ as such did not affect the adhesion of MCL cells to FN, HS-5 or HS-27a (Supplementary Fig. 6). Furthermore, the adhesion of BTKi-sensitive JeKo-1 cells to FN, HS-5 or HS-27a was also inhibited by KIN-8194 treatment in a HCK-dependent manner (Fig. 4A, B). In contrast, whereas ibrutinib treatment also reduced the adhesion of JeKo-1, adhesion of Granta-519 or Maver-1, to either FN, HS-5, or HS-27a, was not affected (Fig. 4B and Supplementary Fig. 5). Finally, the adhesion of all three tested primary MCL samples to fibronectin was also strongly reduced by KIN-8194, whereas ibrutinib only reduced the adhesion of one primary MCL sample (Fig. 4C).

The BCR-BTK signaling pathway plays a key role in the regulation of integrin-mediated adhesion of (malignant) B-cells [23]. Stimulation of the BCR with α-IgM induced integrin-mediated adhesion of JeKo-1 and Maver-1, but not Granta-519, to FN. Importantly, this could be completely repressed by KIN-8194 treatment (Fig. 4D). Overexpression of HCK^T333M^ partially prevented this inhibition of aIgM-induced adhesion, especially at low concentrations of KIN-8194 (Fig. 4D). In line with our previous studies [3, 4, 29], ibrutinib could also abrogate the α-IgM-induced adhesion to FN; however, supporting a prominent role for inhibition of BTK in this effect of ibrutinib, this was not prevented by HCK^T333M^ overexpression (Fig. 4D). Noteworthy, KIN-8194 and ibrutinib treatment did not reduce integrin-α4, -β1 or -β2 levels on the cell membrane and the observed loss of adhesion of JeKo-1, Maver-1, Granta-519, and primary MCL cells could be overcome by stimulation of PKC by PMA (Supplementary Fig. 7). This demonstrates that the observed impaired adhesion is not merely a result of reduced integrin expression or cell viability, but rather of reduced integrin activity and activation.

Taken together, these findings show that KIN-8194 treatment results in a loss of antigen-dependent and –independent adhesion of MCL cells to FN as well as antigen-independent adhesion to stromal cells, and KIN-8194 inhibits the integrin-mediated adhesion of MCL cells which are sensitive to BTK inhibition but also of MCL cells insensitive to BTKi.

### Various modes of acquired BTKi resistance can be overcome by KIN-8194

**Fig. 5:**
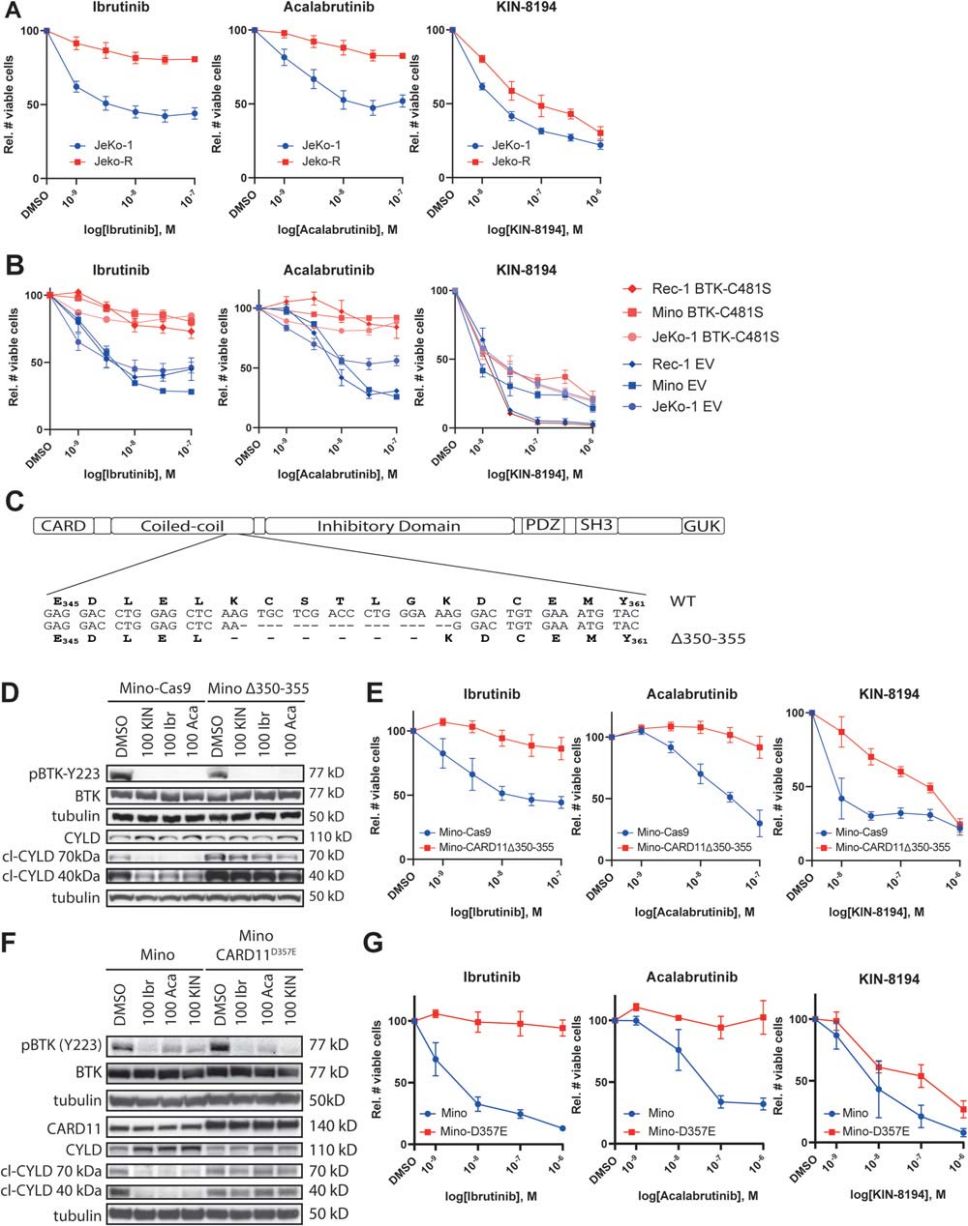
KIN-8194 overcomes secondary resistance to BTKi. **A** Number of viable JeKo-1 or JeKo-R cells following 7 days of treatment with ibrutinib, acalabrutinib, or KIN-8194. **B** Number of viable JeKo-1, Mino, and Maver-1 cells transduced with an LZRS-GFP empty vector (EV) control or LZRS-BTK^C481S^-GFP following 7 days of treatment with various concentrations of ibrutinib, acalabrutinib, or KIN-8194. **C** Schematic representation of CARD11 domains and the amino acids deleted in Mino-Cas9^CARD11-Δ350-355^. **D** Western blot analysis of Mino cells expressing Cas9 (Mino-Cas9) and Mino-Cas9^CARD11-Δ350-355^ (Mino Δ350–355) cells treated for 6 h (P-BTK, BTK, top tubulin) or 24 h (CYLD cleavage, bottom tubulin) with 100 nM ibrutinib, acalabrutinib, or KIN-8194. cl-CYLD indicates MALT1-mediated cleaved CYLD fragments of 70 and 40 kDa. Tubulin serves as a loading control. **E** Number of viable Mino-Cas9 or Mino-Cas9^CARD11-Δ350-355^ cells following 7 days of treatment with ibrutinib, acalabrutinib, or KIN-8194. The number of viable cells was determined by flow cytometric analysis and 7-AAD staining. The number of viable cells was normalized to the DMSO-treated condition. Data are presented as mean ± S.E.M. of at least three independent experiments performed in triplicate. **F** Western blot analysis of Mino cells and Mino cells transduced with pMSCV-CARD11D357E treated for 6 h (P-BTK, BTK, top tubulin) or 24 h (CYLD cleavage, CARD11, bottom tubulin) with 100 nM ibrutinib, acalabrutinib, or KIN-8194. cl-CYLD indicates MALT1-mediated cleaved CYLD fragments of 70 and 40 kDa. Tubulin serves as a loading control. **G** Number of viable Mino cells or Mino cells transduced with pMSCV-CARD11D357E following 7 days of treatment with ibrutinib, acalabrutinib, or KIN-8194. The number of viable cells was determined by flow cytometric analysis and 7-AAD staining. The number of viable cells were normalized to the DMSO-treated condition. Data are presented as mean ± S.E.M. of at least three independent experiments performed in triplicate.

In order to investigate whether KIN-8194 can also inhibit the growth of MCL cells with acquired BTKi resistance, we generated BTKi-resistant JeKo-1 cells (JeKo-R) by prolonged culturing in the presence of ibrutinib. Subsequent culturing of JeKo-R cells in the absence of ibrutinib did not resensitize the cells to ibrutinib or acalabrutinib treatment, indicating that the BTKi resistance was not reversible (data not shown). Furthermore, targeted sequencing did not detect mutations in *BTK* or *PLCG2* that are linked with BTKi resistance (data not shown). Importantly, whereas both ibrutinib and acalabrutinib can no longer reduce the growth of JeKo-R cells, the growth inhibition by KIN-8194 was largely retained (Fig. 5A).

A second mode of BTKi resistance is the acquisition of the BTK^C481S^ mutation. We have previously reported that in ABC-DLBCL and WM, BTK^C481S^ overexpression results in resistance to ibrutinib, but not to KIN-8194 [22]. In line with these findings we show that in MCL, BTK^C481S^ overexpression confers resistance to ibrutinib and acalabrutinib but does not affect the sensitivity to KIN-8194 (Fig. 5B).

Activating mutations in the BCR pathway downstream of BTK, such as in CARD11 or PLCγ2, have also been reported to cause BTKi resistance in MCL [15] as well as DLBCL [30–32]. To investigate how such mutations affect the anti-proliferative potency of KIN-8194, we aimed to generate a D357E gain-of-function point mutation of CARD11 in Mino cells by means of CRISPR-Cas9 mediated knock-in. In this process we created Mino-Cas9 cells with an in-frame deletion of amino acids 350–355 of CARD11 (Fig. 5C). No pathogenic variants of CARD11 have been described involving the deleted amino acids, but they are in the near vicinity of D357 and Y361, which both have been shown to be mutated in MCL and cause ibrutinib resistance in vitro [15]. We show that Mino-Cas9^CARD11Δ350-355^ cells have increased MALT1 protease activity, illustrated by constitutive BTKi-insensitive cleavage of the MALT1 substrate CYLD (Fig. 5D), indicating that the deletion in CARD11 results in a gain-of-function variant with constitutive activation of the CARD11-Bcl10-MALT1 (CBM) complex. In line with this, Mino-Cas9^CARD11Δ350-355^ cells are resistant to ibrutinib and acalabrutinib, although BTK is still inhibited (Fig. 5D, E). Importantly, KIN-8194 retains anti-proliferative capacity in Mino-Cas9^CARD11Δ350-355^ cells although it is reduced compared to control cells (Fig. 5E). Likewise, Mino cells overexpressing a CARD11^D357E^ mutant, which has been identified in MCL patients [15], also combine constitutive activation of the CBM complex with resistance to BTKi, while partially retaining the anti-proliferative capacity of KIN-8194 (Fig. 5F, G). Combined, our data show that KIN-8194 can overcome acquired BTKi resistance, whether this resistance is mutation-driven or through transcriptional reprogramming.

In conclusion, our findings indicate that dual BTK-HCK targeting by KIN-8194 can inhibit the growth of MCL cells, including MCL cells that are BTKi insensitive due to primary or acquired BTKi resistance. Furthermore, the integrin-mediated adhesion of BTKi-sensitive and insensitive MCL cells to FN and stromal cells is also strongly impaired by KIN-8194.

## Discussion

BTK inhibitors have drastically improved MCL patient outcome in recent years. Nevertheless, one-third of patients show primary resistance and acquired resistance inevitably develops [6–8]. Once patients relapse after BTKi treatment they hardly respond to salvage chemotherapy and have very poor outcomes [33, 34]. A common strategy to overcome drug resistance is combining multiple drugs in order to target various pathways. This can, however, lead to unpredictable drug-drug interactions and complex pharmacokinetics have to be taken into account. Dual inhibitors are small molecules designed to inhibit multiple proteins and can thus serve as an alternative to combination treatment.

We have recently shown that the dual inhibitor of BTK and HCK, KIN-8194, potently reduced the viability of ABC-DLBCL and WM cells harboring the pathogenic Myd88-L265P mutation [22]. Furthermore, KIN-8194 had an excellent in vivo pharmacokinetic profile, was well tolerated in mice, and showed clinical efficacy in ABC-DLBCL xenografted mice [22]. Importantly, expression of both BTK and HCK is largely restricted to cells of the hematopoietic lineage, limiting potential adverse effects of KIN-8194 on healthy cells. Since we have previously shown that HCK is an attractive drug target in MCL [21], we investigated whether KIN-8194 could have an added value over BTK inhibitors in preclinical MCL models.

First we showed that KIN-8194 is active in BTKi-sensitive (Rec-1, Mino, and JeKo-1) and BTKi-insensitive (Granta-519, Maver-1) MCL cell lines with a GI50 between 7 and 25 nM (Fig. 1). In mice it has been shown that KIN-8194 is well tolerated at dosages where plasma levels of KIN-8194 reach approximately 2 µM [22]. Only Z-138 did not respond to either KIN-8194 or BTKi. Notably, this cell line represents an atypical MCL cell line with blastoid transformation which lacks HCK expression (Fig. 1C) [21, 25]. Importantly, the ATP-activity of all, and the viability of 4 out of 5 tested primary MCL samples was also strongly diminished by KIN-8194 whereas only modest effects of ibrutinib were seen (Fig. 1E). In order to determine to what extent HCK inhibition contributes to the observed growth inhibition we overexpressed the gatekeeper mutant HCK^T333M^ in Granta-519, JeKo-1, Mino, and Maver-1. In Granta-519 HCK inhibition is responsible for the vast majority of the growth reduction, as ~75-fold higher KIN-8194 concentrations were required to inhibit the growth of HCK^T333M^ overexpressing cells in comparison to the control cells (Fig. 2A). Although in Mino, JeKo-1, and Maver-1 the HCK-mediated growth inhibition is more modest (3- to 5-fold), it is still substantial, indicating that inhibition of HCK as well as other kinases mediates the growth inhibitory effect of KIN-8194 in these cell lines. Western blot analysis of the BTKi-insensitive cell lines Granta-519 and Maver-1 showed that nanomolar concentrations of KIN-8194 are sufficient to completely inhibit phosphorylation of AKT as well as its downstream mediator S6 in a HCK-dependent manner, whereas canonical and non-canonical Nf-κB or MAPK signaling are not affected (Fig. 3 and Supplementary Fig. 3). The PI3K/AKT pathway plays a critical role in promoting cell survival and proliferation and has been implicated in MCL pathogenesis [35–37]. Importantly, primary and secondary resistance of MCL patients to BTKi has previously been linked to activation of the PI3K/AKT axis [9–11, 14, 38].

We show that JeKo-1 cells with acquired resistance to BTKi through prolonged culturing in the presence of ibrutinib (JeKo-R) are still sensitive to KIN-8194 (Fig. 5A). Furthermore, Mino cells rendered BTKi resistant by an in-frame gain-of-function deletion in *CARD11* or by overexpression of a CARD11^D357E^ mutant, previously identified in a MCL patient, were also still sensitive to KIN-8194 (Fig. 5D–G). Notably, the DLBCL cell line OCI-Ly3, which also harbors a gain-of-function CARD11 mutation, is insensitive to BTKi as well as KIN-8194 [22]. The JeKo-R, Mino-Cas9^CARD11-Δ350-355^, and Mino-CARD11^D357E^ cells did show a partial decrease in KIN-8194-induced growth inhibition (Fig. 5A, E). A likely explanation is that whereas BTK inhibition by KIN-8194 no longer affects the growth of JeKo-R, Mino-Cas9^CARD11-Δ350-355^, or Mino-CARD11^D357E^ cells, inhibition of other kinases, including HCK, still does. This is substantiated by our finding that inhibition of multiple kinases by KIN-8194, including HCK, is responsible for the growth reduction observed in the parental JeKo-1 and Mino cells (Fig. 2). In line with these results, we observed that also overexpression of BTK^C481S^, a mutation resulting in loss of BTKi binding recurrently found in patients with acquired BTKi resistance, overcomes BTKi sensitivity without affecting KIN-8194 potency in MCL cells (Fig. 5B) [22].

The clinical efficacy of BTKi in MCL critically depends on the egress of MCL cells from the protective tumor microenvironment into the peripheral blood, by inhibition of their integrin-mediated adhesion [2–4]. We have previously shown that genetic perturbation of HCK in MCL results in inhibition of integrin α4-mediated adhesion to FN or HS-27a [21]. Extending on this observation, we now show that KIN-8194 inhibits the integrin-mediated adhesion of BTKi-sensitive and insensitive MCL to FN and stromal cells in an HCK-dependent manner (Fig. 4). In contrast, ibrutinib only reduces the adhesion of BTKi-sensitive MCL cells. Furthermore, we show that KIN-8194 also inhibits BCR-controlled adhesion of MCL cells to FN in an HCK-dependent manner. Overexpression of HCK^T333M^ cannot completely rescue the KIN-8194-evoked reduction in aIgM-stimulated adhesion, since higher KIN-8194 concentrations will inhibit BTK and BTK inhibition also reduces BCR-controlled adhesion of MCL cells to FN (Fig. 4D). Our data indicate that KIN-8194 can inhibit integrin-mediated adhesion of MCL cells via the BCR-BTK pathway, as well as via a second, BTK-independent and HCK-dependent, mechanism.

Taken together, our results show that the dual HCK/BTK inhibitor KIN-8194 potently inhibits the growth and integrin-mediated adhesion of MCL cells, independent of their sensitivity to BTKi, at in vivo relevant concentrations. Importantly, we show that KIN-8194 is also effective in MCL cells with primary and acquired resistance to BTKi. Since KIN-8194 exerts these anti-tumorigenic effects through inhibition of multiple kinases, this will minimize the chance of resistance to KIN-8194. These results encourage the further clinical development of KIN-8194 as a potential treatment for MCL patients.

### Supplementary information

Supplemental Material
