# Supplementary material for: The dual HCK/BTK inhibitor KIN-8194 impairs growth and integrin-mediated adhesion of BTKi-resistant mantle cell lymphoma
Source: Leukemia. 2024 Mar 7;38(7):1570–80. doi: 10.1038/s41375-024-02207-9 (PMC11216997; doi:10.1038/s41375-024-02207-9)
Supplement: Supplementary file 1 — Supplemental Material [file 41375_2024_2207_MOESM1_ESM.pdf]

Supplementary Figure 1

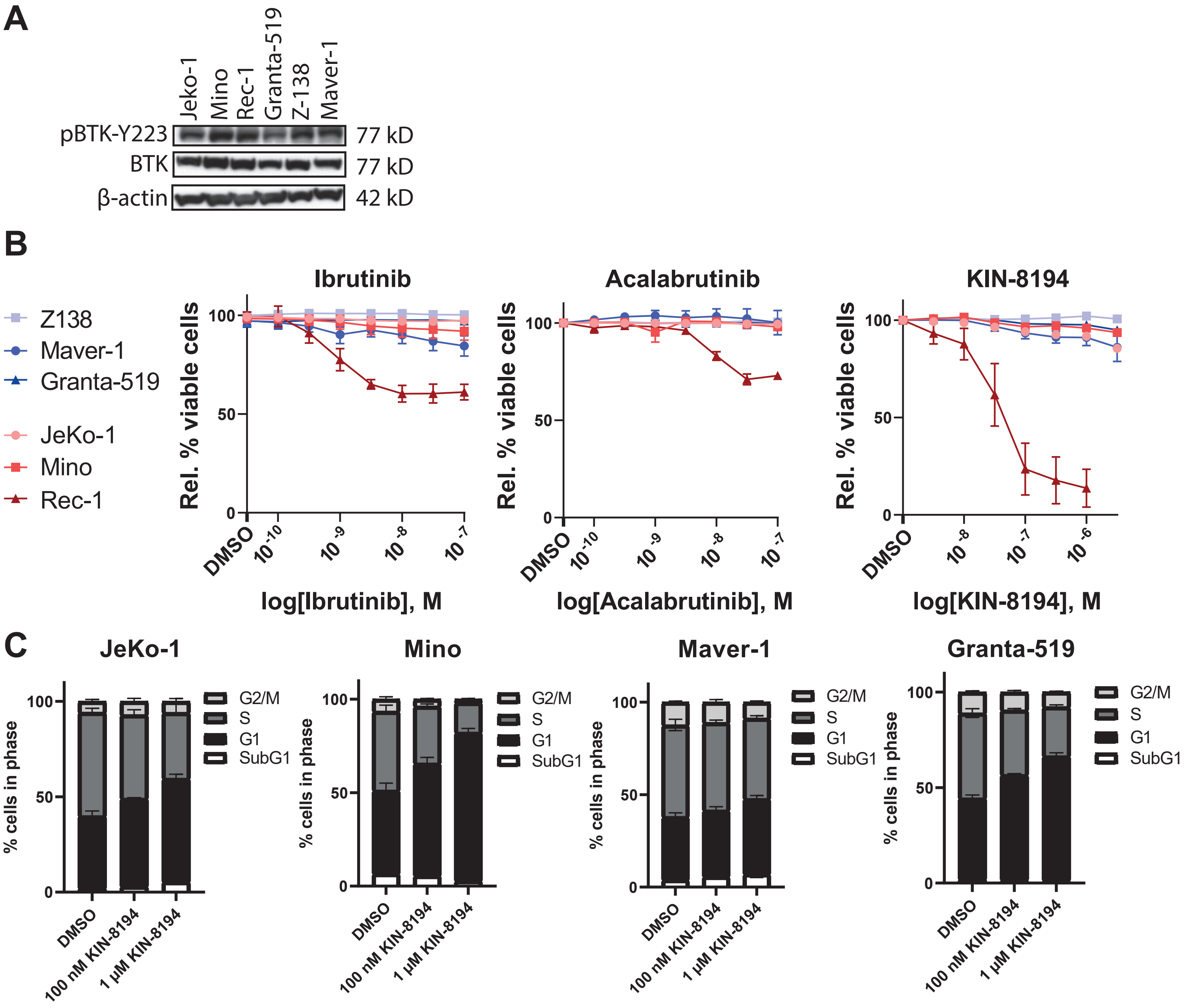

Supplementary Figure 1: Ibrutinib, acalabrutinib and KIN-8194 represses the proliferation of MCL cell lines while only affecting the viability of Rec-1 cells

(A) Western blot analysis of BTK phosphorylation (Y223) in MCL cell lines. β-actin serves as a loading control. (B) Percentage of viable MCL cells following 7 days of treatment with ibrutinib, acalabrutinib of KIN-8194. Cell viability was determined by flow cytometric analysis and 7-AAD staining. The percentage of viable cells was normalized to the DMSO treated condition. Data presented as mean +/- S.E.M. of at least three independent experiments performed in triplicate. (C) Cell cycle analysis after KIN-8194 treatment. The percentage of cells in Sub-G1 (BrdU-, <To-Pro-3-), G1 (BrdU-,To-Pro-3-), S (BrdU+), and G2 (BrdU+, To-Pro-3+) was determined by flow cytometric analysis after 4 days of treatment with the indicated concentrations of KIN-8194. Data presented as mean +/- S.E.M. of three independent experiments.

Supplementary Figure 2

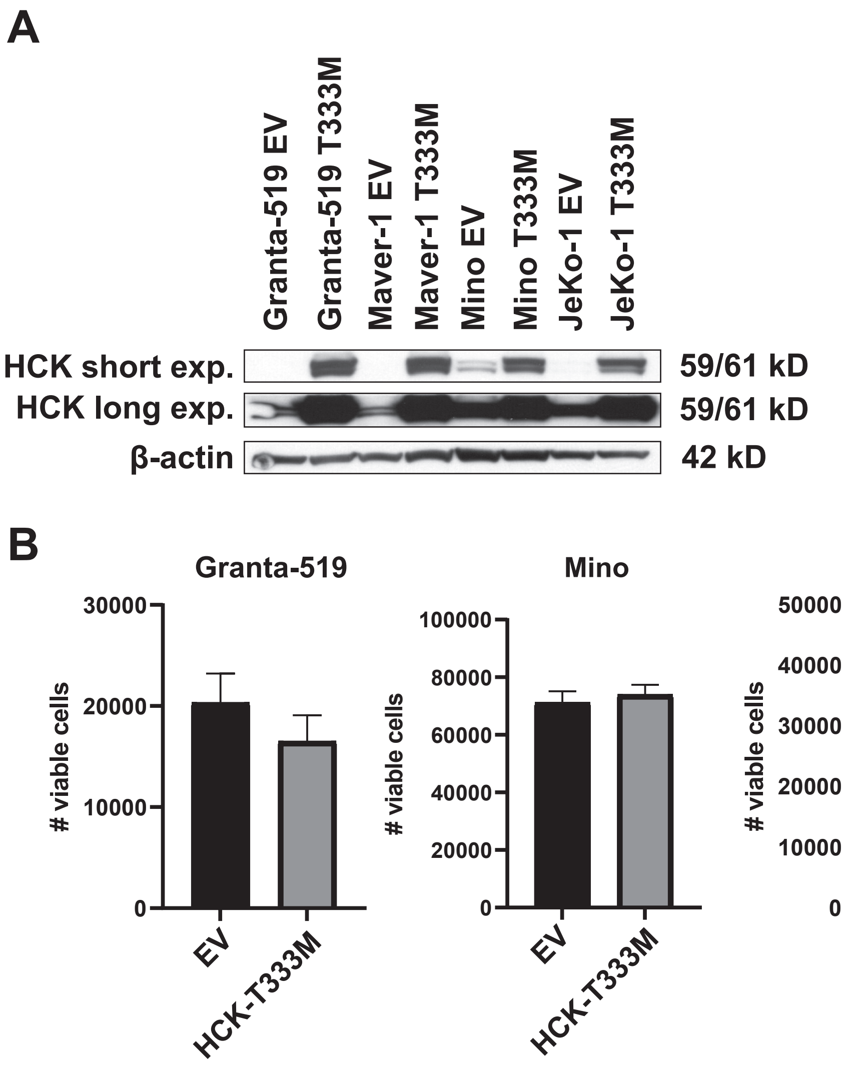

Supplementary Figure 2: HCK-T333M overexpression

(A) Western blot analysis of Granta-519, Maver-1, Mino and JeKo-1 cells transduced with an LZRS-GFP empty vector (EV) control or LZRS-HCK^{T333M}-GFP. β-actin serves as a loading control. A long exposure of the same blot is also presented to show expression of endogenous HCK (please also see Figure 1C and ref. 21) (B) Number of viable Granta-519, Mino, JeKo-1 or Maver-1 cells transduced with an LZRS-GFP empty vector (EV) control or LZRS-HCK^{T333M}-GFP after 7 days of treatment with 0.01 % DMSO (control). Data presented as mean +/- S.E.M. of three independent experiments performed in triplicate.

Supplementary Figure 3

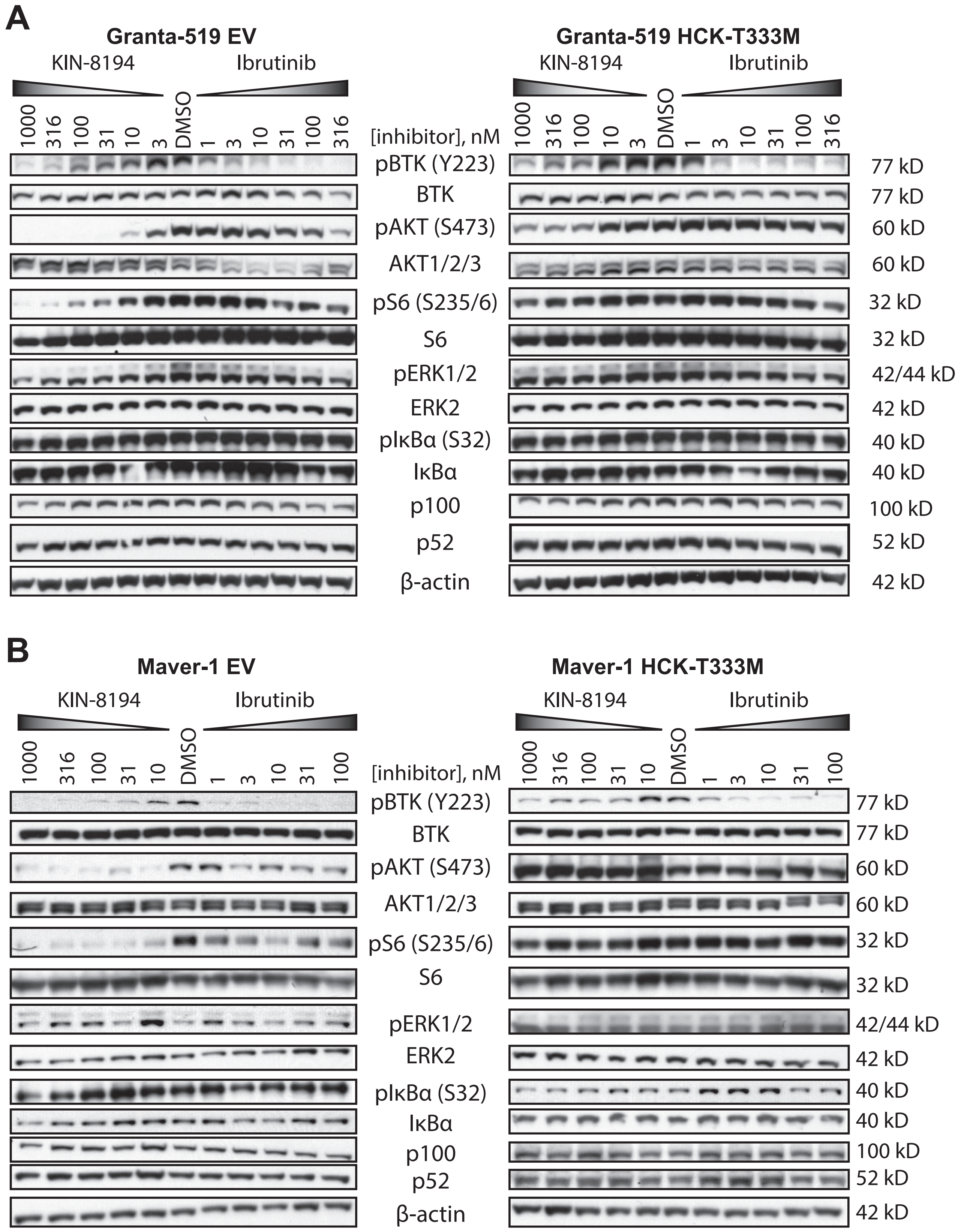

Supplementary Figure 3: Nanomolar concentrations of KIN-8194, but not ibrutinib, inhibit AKT and S6 phoshorylation in an HCK-dependent manner in BTKi insensitive cells

Western blot analysis of Granta-519 (A) and Maver-1 (B) cells transduced with an LZRS-GFP empty vector (EV) control or LZRS-HCK^{T333M}-GFP treated with various concentrations of ibrutinib or KIN-8194 for 6 hours. β-actin serves as a loading control.

Supplementary Figure 4

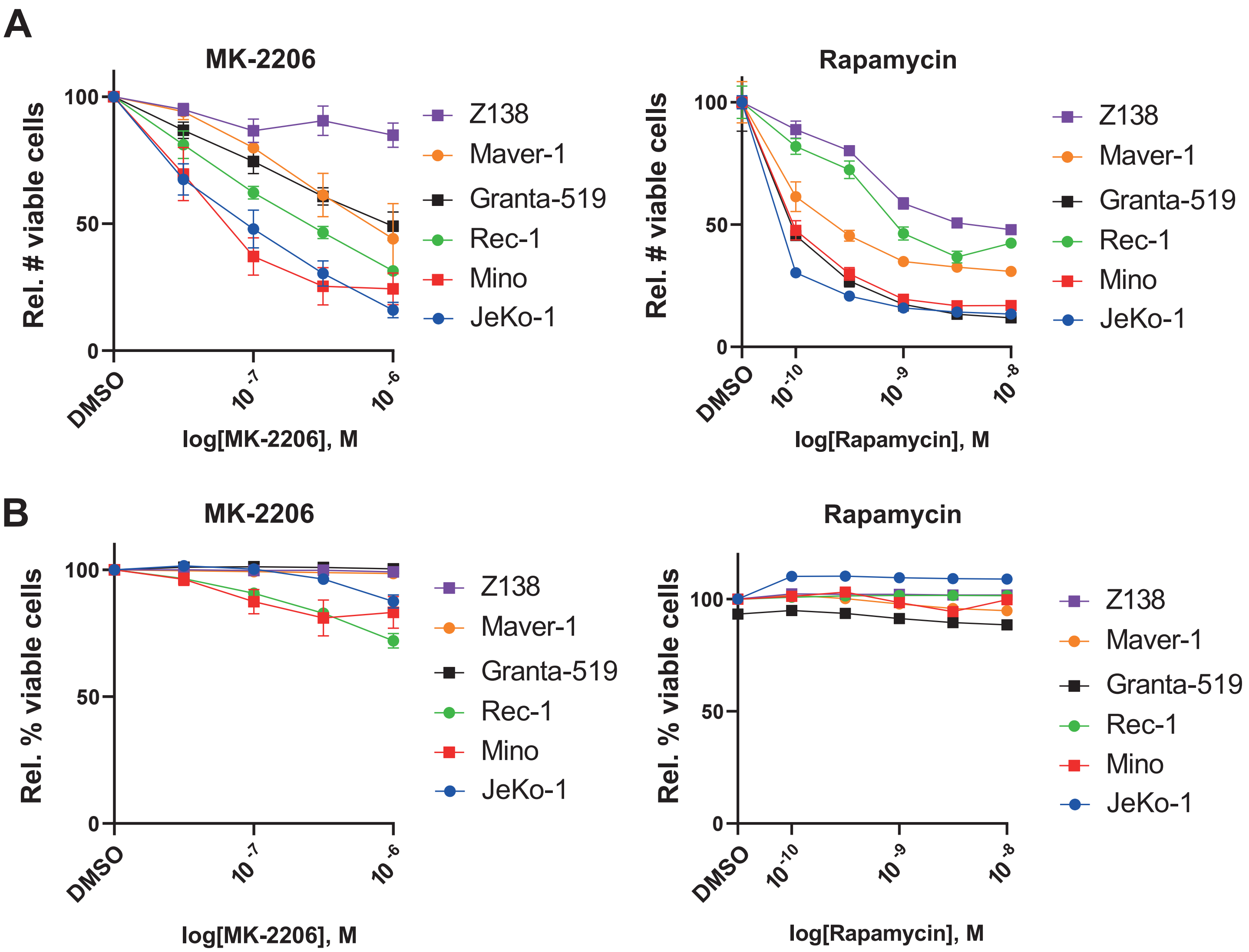

Supplementary Figure 4: AKT and mTOR inhibition reduces the growth of MCL cell lines without affecting the cell viability

Number of viable MCL cells, determined by flow cytometric analysis and 7-AAD staining, following 7 days of treatment with MK-2206 or rapamycin (A). Percentage of viable MCL cells, determined by flow cytometric analysis and 7-AAD staining, following 7 days of treatment with MK-2206 or rapamycin (B). The number and percentage of viable cells were normalized to the DMSO treated condition.

Supplementary Figure 5

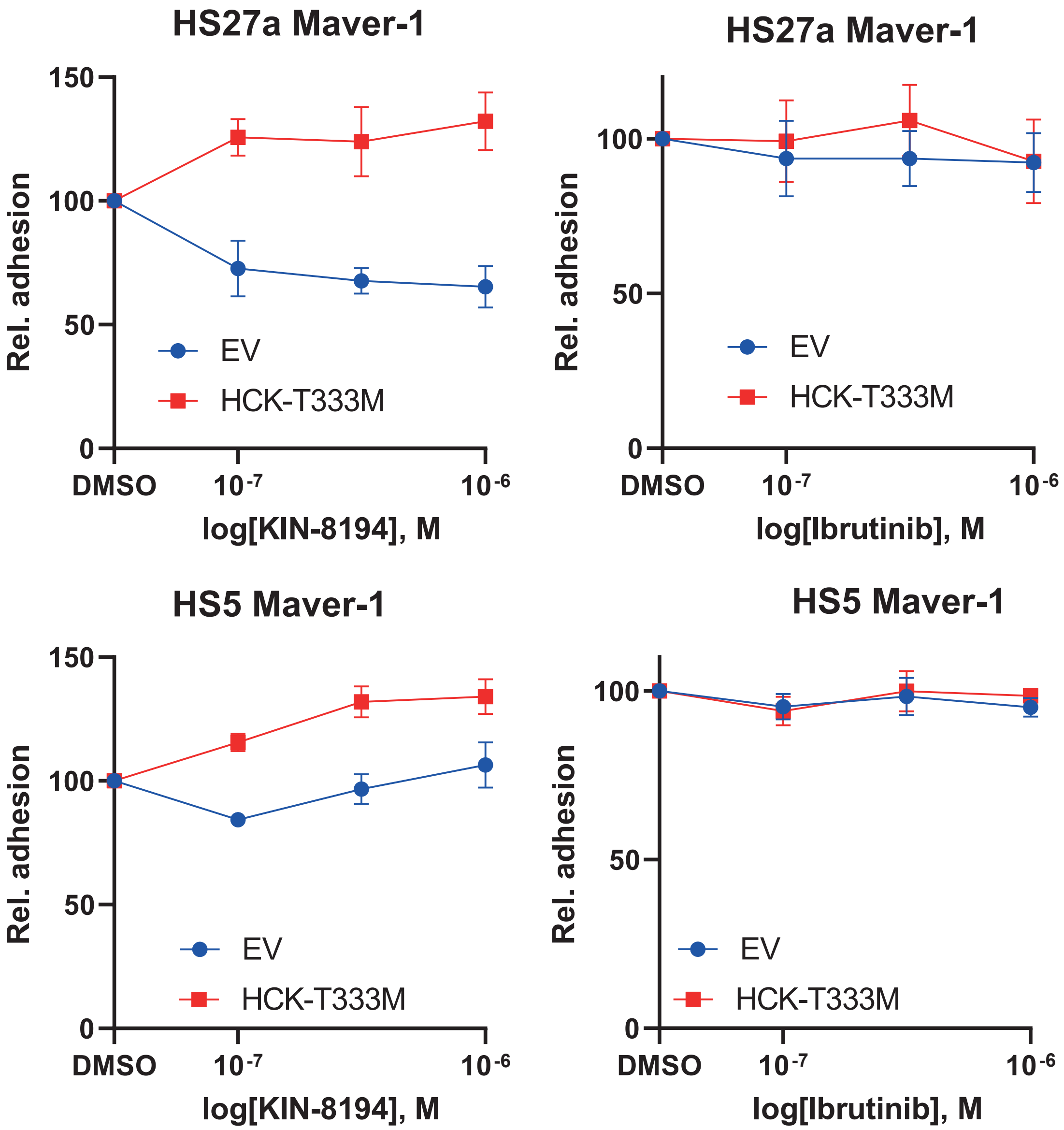

Supplementary Figure 5: KIN-8194 inhibits the adhesion of Maver-1 to HS27a

Maver-1 cells were transduced with an LZRS-GFP empty vector (EV) control or LZRS-HCK^{T333M}-GFP. Cells were treated for 30 minutes with various concentrations of KIN-8194 or ibrutinib and allowed to adhere for 30 minutes. Non-adherent cells were removed by washing. The ratio of MCL cells to stromal cells was determined. The percentage of adhesion was normalized to the untreated control. Data presented as mean +/- S.E.M. of at least two independent experiments performed in triplicate.

Supplementary Figure 6

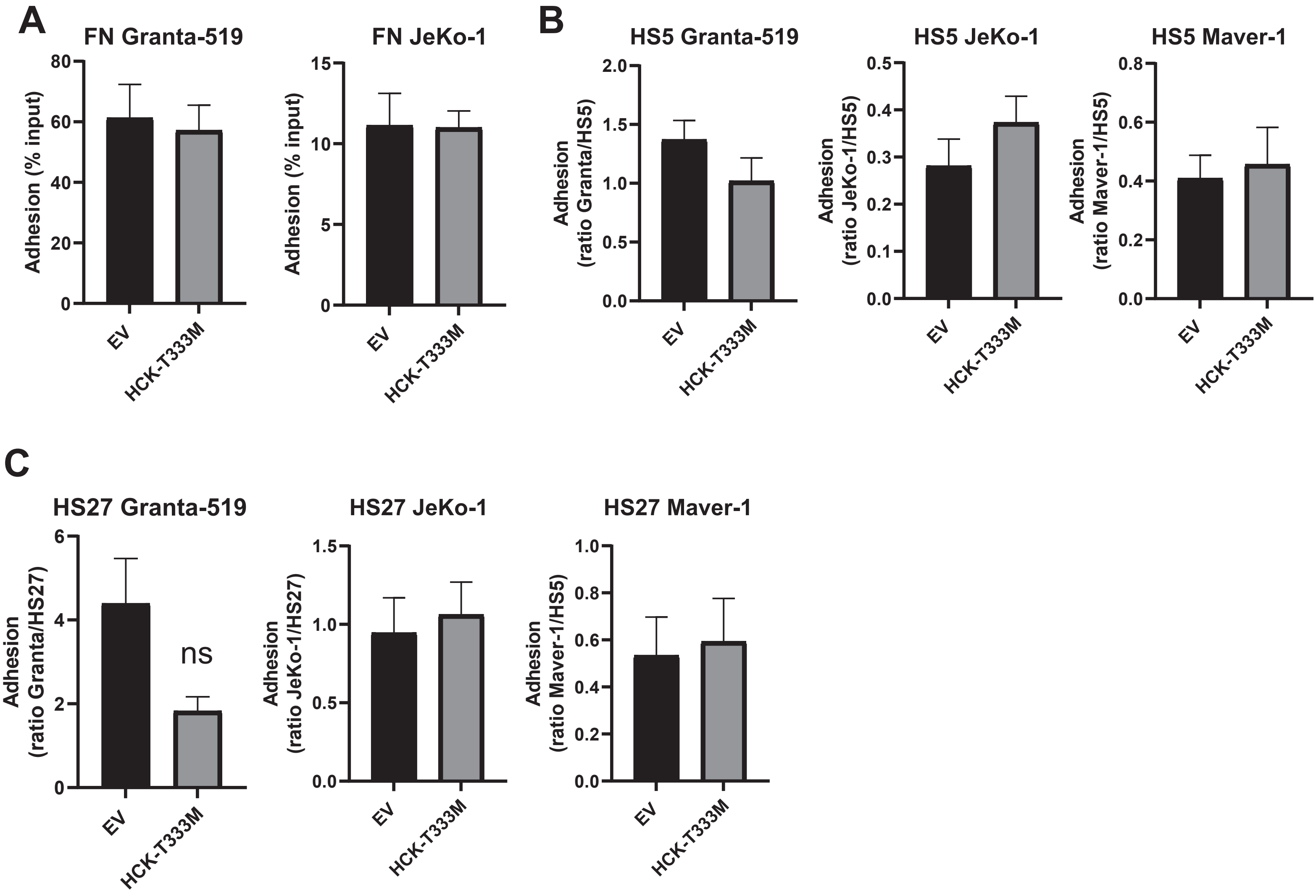

Supplementary Figure 6: HCK^{T333M} overexpression does not affect cell adhesion

(A) Adhesion of Granta-519 and JeKo-1 cells transduced with an LZRS-GFP empty vector (EV) control or LZRS-HCK^{T333M}-GFP to fibronectin. Adhesion is shown as percentage of total input. (B) Adhesion of Granta-519, JeKo-1 and Maver-1 cells transduced with an LZRS-GFP empty vector (EV) control or LZRS-HCK^{T333M}-GFP to HS5 stromal cells. Adhesion is shown as the ratio of MCL cells over stromal cells in order to correct for loss of stromal cells. (C) Adhesion of Granta-519, JeKo-1 and Maver-1 cells transduced with an LZRS-GFP empty vector (EV) control or LZRS-HCK^{T333M}-GFP to HS27a stromal cells. Adhesion is shown as the ratio of MCL cells over stromal cells in order to correct for loss of stromal cells. Data are presented as mean +/- S.E.M. of at two (Maver-1) or three (Granta-519 and JeKo-1) independent experiments performed in triplicate. ns indicates non-significant difference ($p=0.129$) according to unpaired t-test with Welch’s correction.

Supplementary Figure 7

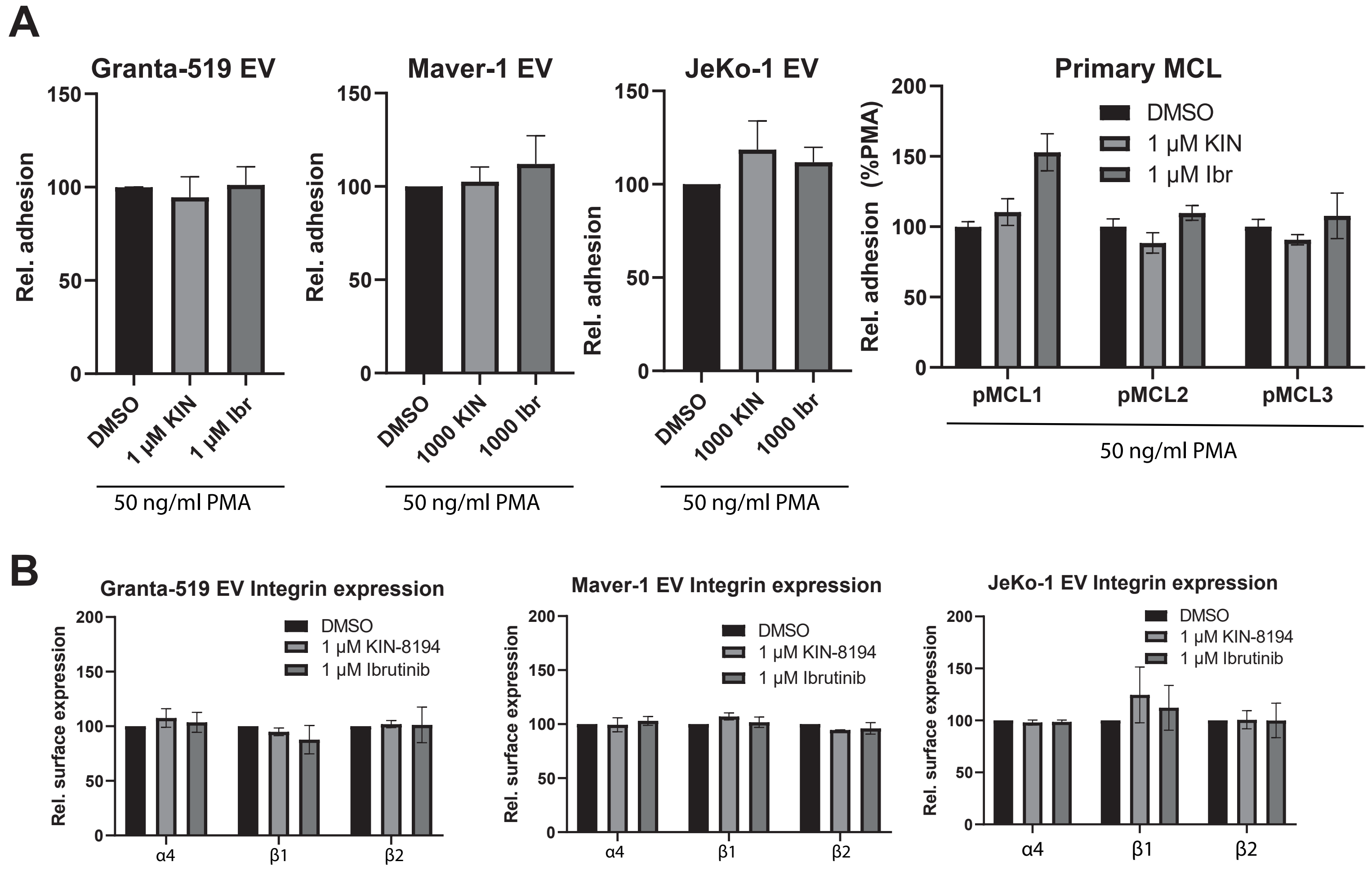

Supplementary Figure 7: Loss of adhesion of MCL cells treated with KIN-8194 or ibrutinib does not reflect a loss of cell viability or integrin expression

(A) Adhesion of Granta-519, Maver-1, JeKo-1 transduced with an LZRS-GFP empty vector (EV) control and of three primary MCL samples after 30 minutes treatment with 1 µM KIN-8194 or ibrutinib and allowed to adhere to fibronectin coated plates in the presence of 50 ng/ml PMA. Non-adherent cells were removed by washing. Percentage of adherent cells were normalized to the untreated control. Data presented as mean +/- S.E.M. of at least three independent experiments performed in triplicate with the exception of the data concerning primary MCL samples. (B) Flow cytometric analysis of integrin α4, β1 and β2 on Granta-519, Maver-1 and JeKo-1 cells transduced with an LZRS-GFP empty vector (EV) control and treated for 30 minutes with 1 µM KIN-8194 or ibrutinib. Data presented as mean +/- S.E.M. of three independent experiments.
